# Sequential administration of sclerostin antibody and parathyroid hormone differentially modulates fracture healing in a murine tibial osteotomy model

**DOI:** 10.1371/journal.pone.0354181

**Published:** 2026-07-17

**Authors:** Atsushi Mihara, Kiminori Yukata, Norihiro Nishida, Tetsuya Seto, Ryuta Iwanaga, Kenzo Fujii, Kazuya Uehara, Yuji Saeki, Junji Ohgi, Shunya Tsuji, Masataka Asagiri, Takashi Sakai

**Affiliations:** 1 Department of Orthopaedic Surgery, Yamaguchi University Graduate School of Medicine, Ube, Yamaguchi, Japan; 2 Faculty of Engineering, Yamaguchi University, Ube, Yamaguchi, Japan; 3 Department of Pharmacology, Yamaguchi University Graduate School of Medicine, Ube, Yamaguchi, Japan; Tokushima University, JAPAN

## Abstract

Anabolic agents such as parathyroid hormone (PTH) analogs and sclerostin antibody (Scl-Ab) enhance fracture healing through distinct mechanisms, but whether the order of administration affects healing remains unclear. In a murine tibial osteotomy model, we compared PTH monotherapy (40 μg/kg), Scl-Ab monotherapy (25 mg/kg), and two sequential regimens with matched treatment duration and cumulative exposure between sequential groups: PTH followed by Scl-Ab (PTH → Scl-Ab) and Scl-Ab followed by PTH (Scl-Ab → PTH). Treatment was started on postoperative day 1, and the sequential groups changed agents after 1 week. PTH monotherapy significantly increased mineralized callus volume, whereas Scl-Ab monotherapy produced higher callus volumetric bone mineral density (vBMD) and greater mechanical strength. Among the sequential regimens, Scl-Ab → PTH resulted in significantly higher callus vBMD and ultimate load to failure than PTH → Scl-Ab, despite comparable callus volumes. Histological analysis showed a higher proportion of cartilaginous tissue in the PTH monotherapy and PTH → Scl-Ab groups, suggesting persistence of an earlier phase of endochondral ossification. These findings indicate that early Scl-Ab administration followed by PTH promotes earlier callus mineralization and mechanical recovery than the reverse sequence in this murine model. Although the study was limited primarily to early healing time points, the results suggest that the temporal sequence of anabolic signaling, rather than agent selection alone, can influence early fracture repair.

## Introduction

Fracture healing is a highly orchestrated biological process involving the coordinated activation of inflammation, chondrogenic, and osteogenic pathways. Among the signaling pathways implicated in this process, canonical Wnt/*β*-catenin signaling plays a pivotal role in skeletal development, bone remodeling, and fracture repair. Previous studies have demonstrated that Wnt-related genes are upregulated at fracture sites, with peak activation occurring approximately 7–14 days after injury in murine and rat models, suggesting a critical temporal role for canonical Wnt/*β*-catenin signaling during fracture healing [1,2].

Sclerostin, a glycoprotein predominantly produced by osteocytes, is a potent endogenous antagonist of Wnt signaling and a key negative regulator of bone formation. Pharmacological inhibition of sclerostin using a sclerostin antibody (Scl-Ab) activates canonical Wnt/*β*-catenin signaling and induces robust anabolic effects on bone. Scl-Ab enhances bone mass and strength through a modeling-based mechanism characterized by increased osteoblastic activity and concomitant suppression of bone resorption [3,4]. Several animal studies have reported favorable effects of Scl-Ab on fracture healing [5]. In clinical settings, however, randomized clinical trials have not demonstrated a clear benefit of Scl-Ab for accelerating fracture healing in humans [6,7]. This discrepancy highlights the need for a more refined understanding of how and when this agent should be applied during the fracture repair process.

Parathyroid hormone (PTH) analogs represent another class of anabolic agents with well-documented effects on fracture healing. Both experimental and clinical studies have shown that intermittent PTH administration accelerates fracture repair at various skeletal sites, including the femur, vertebrae, and distal radius [8–15]. Unlike Scl-Ab, PTH exerts its anabolic effects through a remodeling-based mechanism, stimulating both bone formation and resorption and thereby increasing overall bone turnover [3,16]. PTH also strongly promotes the proliferation and early differentiation of mesenchymal stem cells and osteoprogenitor cells [16], resulting in sustained elevations of bone formation markers during continued treatment. These distinct biological profiles indicate that, although both PTH and Scl-Ab are categorized as anabolic agents, they act on different stages of the osteoblast lineage and influence fracture healing through fundamentally different mechanisms [3].

These mechanistic differences provide a rationale for sequential anabolic therapy. In osteoporosis treatment, clinical studies have shown that switching between anabolic agents produces different skeletal responses depending on the order of administration, suggesting that the sequence of anabolic signaling is biologically meaningful [17,18]. In contrast, fracture healing is a time-dependent regenerative process governed by tightly regulated phases of cellular proliferation, lineage commitment, and mineralization. Therefore, the optimal order of anabolic stimulation during fracture repair may differ from that used in osteoporosis treatment. To date, experimental evidence directly addressing whether the order of PTH and Scl-Ab administration influences fracture healing outcomes remains limited.

The relevance of treatment sequence is further supported by previous findings showing that PTH administration significantly increases the expression of the SOST gene, which encodes sclerostin, at fracture sites [10]. This raises the possibility that PTH-induced upregulation of sclerostin may alter subsequent responsiveness to Scl-Ab. Conversely, early activation of Wnt/β-catenin signaling by Scl-Ab may influence the fate determination of immature osteoprogenitor cells, potentially affecting the balance between osteoblastic and chondrogenic differentiation during fracture repair [2]. Thus, it remains unclear whether PTH should be administered first to expand the progenitor pool, or whether early Scl-Ab treatment should be prioritized to promote osteoblastic lineage commitment.

The purpose of this study was to directly compare the effects of PTH-first and Scl-Ab–first sequential anabolic therapy on fracture healing under strictly controlled conditions using identical agents, doses, and treatment durations. By isolating treatment sequence as the primary variable, we aimed to determine whether the temporal coordination of anabolic signaling critically influences early fracture repair.

## Materials and methods

### Experimental animals

All experimental procedures were conducted in accordance with the National Institutes of Health Guidelines for the Care and Use of Laboratory Animals and were approved by the Institutional Animal Care and Use Committee of our institution (approval number: 35-001). Eight-week-old female wild-type C57BL/6J mice were purchased from Japan SLC, Inc. (Shizuoka, Japan). Mice were housed under standard laboratory conditions with a 12-hour light/dark cycle and were allowed unrestricted access to food and water throughout the experimental period.

### Tibial osteotomy model

A standardized open tibial osteotomy model was established as previously described [10]. Briefly, mice were anesthetized with inhaled isoflurane, and all efforts were made to minimize suffering. A 4–5-mm longitudinal skin incision was made over the anterior aspect of the right tibia. A small entry hole was created at the tibial plateau using a 26-gauge needle to access the medullary cavity. A transverse osteotomy was then performed at the proximal diaphysis of the tibia using a No. 11 scalpel blade. The fibula was left intact.

Fracture stabilization was achieved by inserting a 26-gauge Quincke-type spinal needle (Unisis Co., Tokyo, Japan) into the intramedullary canal from the knee joint. The wound was closed with 5-0 nylon sutures. Postoperatively, mice were allowed unrestricted weight bearing without external immobilization.

### Treatment groups and drug administration

Mice were randomly allocated into five treatment groups: Control, PTH, Scl-Ab, PTH → Scl-Ab, and Scl-Ab → PTH. In the Control group, mice received normal saline for 14 days. In the PTH group, mice received PTH (1–34) for 14 days. In the Scl-Ab group, mice received sclerostin antibody for 14 days. In the PTH → Scl-Ab group, mice received PTH (1–34) during the first 7 days, followed by Scl-Ab during the subsequent 7 days. In the Scl-Ab → PTH group, mice received Scl-Ab during the first 7 days, followed by PTH (1–34) during the subsequent 7 days.

At the 2-week primary endpoint, eight mice per group were used for micro-CT and biomechanical testing, and five mice per group were used for histological analysis. Additional cohorts included five mice per group for histological analysis at 1 week and eight mice per group for micro-CT and biomechanical assessment at 3 weeks.

PTH (Teriparatide BS Subcutaneous Injection Kit; Mochida Pharmaceutical Co., Tokyo, Japan) was administered subcutaneously once daily at a dose of 40 μg/kg. Scl-Ab (romosozumab; Amgen Inc., USA) was administered subcutaneously twice weekly at a dose of 25 mg/kg. Drug administration was started on postoperative day 1 and continued until postoperative day 14. The total treatment duration and cumulative exposure to each agent were matched between the two sequential therapy groups, allowing direct assessment of the effect of administration order.

For the primary endpoint, mice were euthanized on postoperative day 14 after completion of the treatment protocol. The fractured tibiae and contralateral non-fractured tibiae were harvested after removal of the intramedullary pin. The 2-week time point was selected because the sequential therapy groups completed both the first and second treatment phases by postoperative day 14. Previous studies have also shown that fracture healing in mice progresses substantially within approximately 2 weeks after treatment with either PTH or Scl-Ab [5,10], supporting this time point for evaluating early fracture repair.

**Fig 1:**
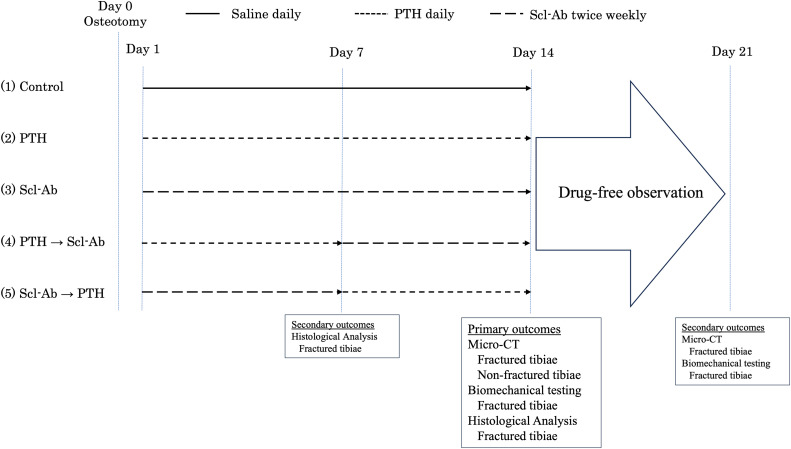
Schematic illustration of the drug administration and evaluation schedule for each experimental group.

To assess later-stage healing, a separate cohort of mice was observed for an additional 1-week drug-free period after completion of the 2-week treatment protocol and euthanized 3 weeks postoperatively. In this cohort, only fractured tibiae were harvested for analysis. The treatment and evaluation schedule is shown in Fig 1.

The sample size was determined based on previous studies using similar murine fracture-healing models and was selected to detect treatment-related differences while minimizing the number of animals used. No formal a priori sample size calculation was performed. Animals were allocated to each group to achieve comparable group sizes for statistical comparison among treatment regimens.

### Micro–computed tomography

Eight specimens per group were subjected to micro–computed tomography (micro-CT) analysis. For the 2-week primary endpoint, both fractured tibiae and contralateral non-fractured tibiae were analyzed. For the 3-week observation cohort, only fractured tibiae were analyzed.

Micro-CT scanning was performed using the CosmoScan GX II system (Rigaku HD Co., Tokyo, Japan) at an isotropic voxel resolution of 2.3 μm. Scanning parameters included a voltage of 90 kV, a current of 88 μA, and a scan time of 4 minutes. Hydroxyapatite phantoms were scanned concurrently for calibration.

For the fractured tibiae, volume of the mineralized external callus and volumetric bone mineral density (vBMD) of the mineralized external callus at the fracture site were quantified. The volume of interest was defined as the entire mineralized external callus throughout the fracture site. For the non-fractured tibiae, trabecular bone volume fraction (BV/TV) and vBMD were quantified in the proximal tibia. The volume of interest was defined as the region beginning 0.3 mm distal to the proximal growth plate and extending 0.5 mm distally. Measurements were performed using Analyze 14.0 software (AnalyzeDirect Inc., Overland Park, KS, USA), as previously described [19,20].

### Biomechanical testing

After micro-CT scanning, the biomechanical properties of fractured tibiae were evaluated using a three-point bending test (EZ-LX 5KN; Shimadzu Co., Kyoto, Japan). Specimens were positioned with a span length of 10 mm, and load was applied vertically at the fracture site at a constant displacement rate of 5 mm/min until failure. Ultimate load to failure and loading stiffness were calculated from the load–displacement curves. One specimen each in the Control and PTH groups was lost due to experimental failure.

### Histological and histomorphometric analysis

Harvested fractured tibiae were fixed in 10% neutral buffered formalin for 3 days. The specimens were then decalcified, dehydrated, embedded in paraffin, and sectioned. Alcian blue hematoxylin/orange G staining was performed to visualize cartilage and bone, respectively.

Histomorphometric analysis was conducted using ImageJ software. The sagittal section closest to the center of the tibia was selected for measurement. The external callus area at the fracture site was manually traced, excluding the original cortical bone and internal callus tissue. For each specimen, the total external callus area and the proportion of cartilage tissue within the external callus were evaluated [21].

Five specimens per group were subjected to histological and histomorphometric evaluation at postoperative week 2 for the primary outcome. Additional specimens were evaluated at postoperative week 1 to assess early histological changes during the initial phase of fracture repair.

### Statistical analysis

Statistical analyses were performed using EZR (version 1.50) [22]. One-way ANOVA followed by Tukey’s post hoc test was used for multiple comparisons. Data are presented as mean ± standard deviation, and a *p* < 0.05 was considered statistically significant.

## Results

### Micro-computed tomography analysis

**Fig 2:**
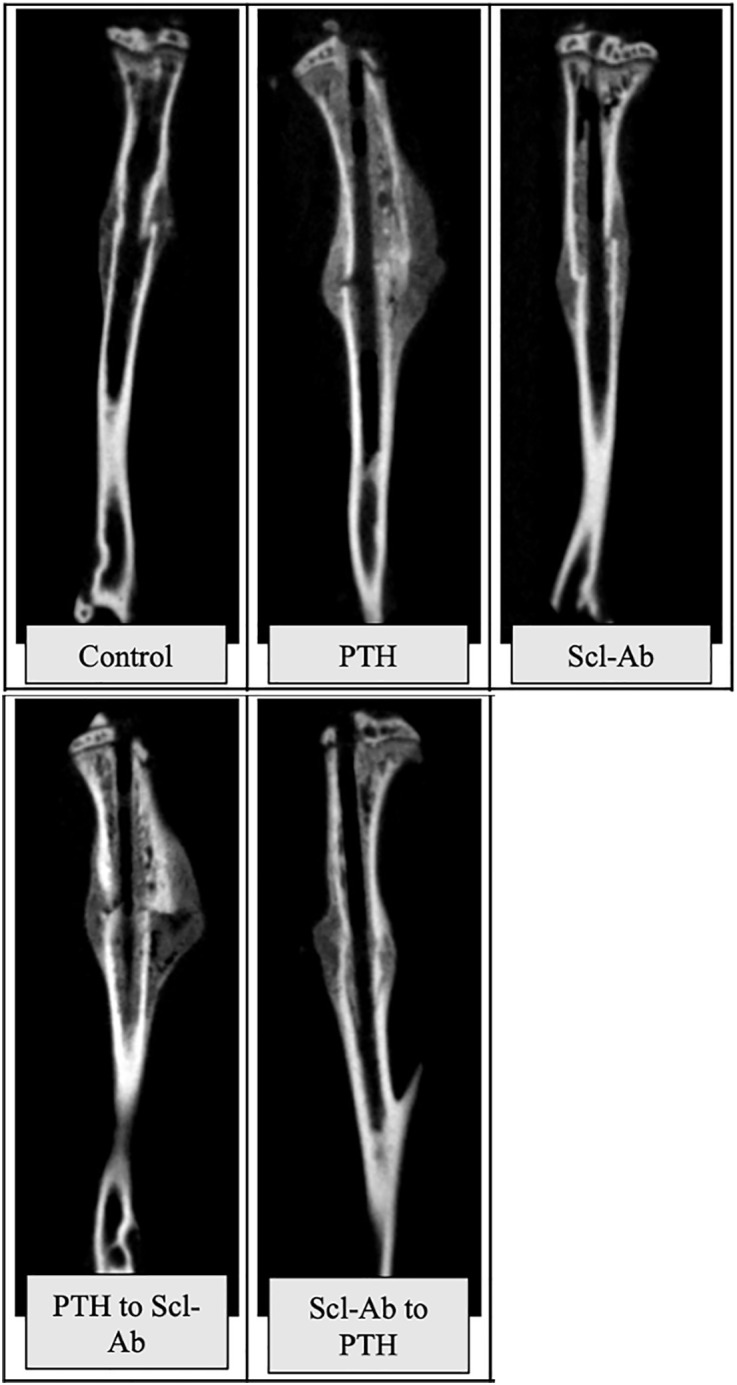
Representative micro–computed tomography images of fracture sites in each group.

**Fig 3:**
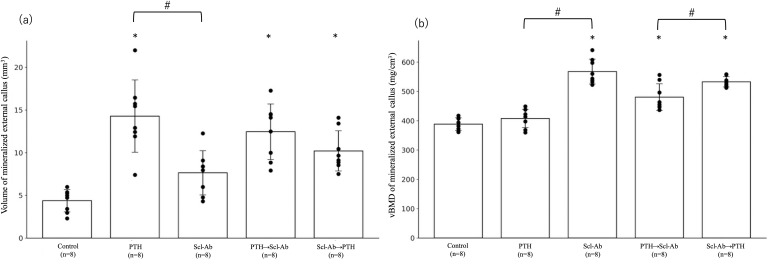
Micro–computed tomography analysis of fracture callus. (a) Volume of mineralized external callus at the fracture site. (b) Volumetric bone mineral density of the mineralized external callus. * p < 0.05 compared with the Control group; # p < 0.05 between the indicated groups.

For fractured tibiae, PTH monotherapy resulted in a significant increase in mineralized external callus volume compared with the Control group on postoperative day 14, whereas Scl-Ab monotherapy did not. In contrast, callus vBMD was significantly higher in the Scl-Ab monotherapy group than in the PTH monotherapy group. Among the sequential therapy groups, callus vBMD was significantly higher in the Scl-Ab → PTH group than in the PTH → Scl-Ab group, although callus volume did not differ significantly between the two groups (Figs 2 and 3). At the 3-week endpoint, callus vBMD showed a similar pattern among groups, with increased absolute vBMD values in all groups compared with those at 2 weeks (S1 Fig). These findings suggest that PTH predominantly promotes callus expansion, whereas Scl-Ab enhances callus mineralization, and that early administration of Scl-Ab may accelerate maturation of the fracture callus.

**Fig 4:**
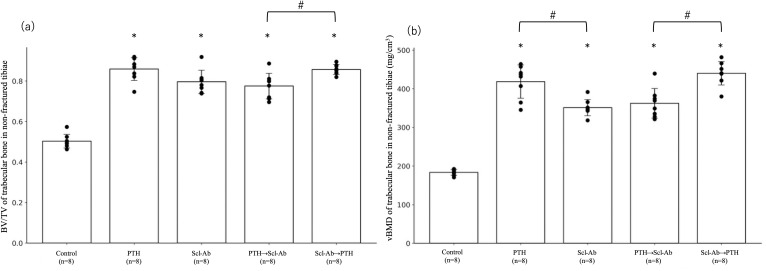
Micro–computed tomography analysis of trabecular bone in non-fractured tibiae. (a) Trabecular bone volume fraction in the proximal tibia. (b) Volumetric bone mineral density of trabecular bone in the proximal tibia. * p < 0.05 compared with the Control group; # p < 0.05 between the indicated groups.

For non-fractured tibiae, all treatment groups showed significant increases in trabecular BV/TV and vBMD compared with the Control group. Among the sequential therapy groups, both BV/TV and vBMD were significantly higher in the Scl-Ab → PTH group than in the PTH → Scl-Ab group. Among the monotherapy groups, vBMD was significantly higher in the PTH group than in the Scl-Ab group (Fig 4).

### Biomechanical testing

**Fig 5:**
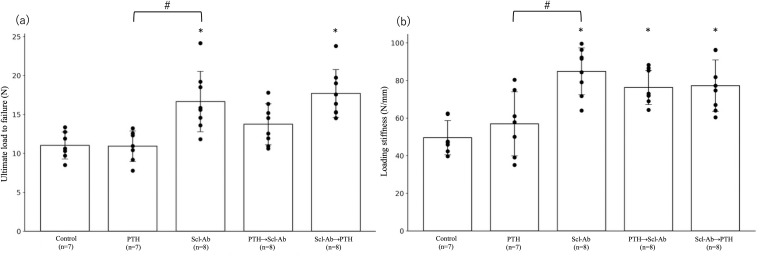
Biomechanical assessment by three-point bending test. (a) Ultimate load to failure. (b) Loading stiffness. * p < 0.05 compared with the Control group; # p < 0.05 between the indicated groups.

Biomechanical testing showed that Scl-Ab monotherapy significantly increased ultimate load to failure and loading stiffness compared with PTH monotherapy. Among the sequential therapy groups, the Scl-Ab → PTH regimen resulted in a significantly higher ultimate load to failure than the PTH → Scl-Ab regimen, indicating that treatment sequence influenced early functional recovery of the fracture site (Fig 5). At the 3-week endpoint, ultimate load to failure showed a pattern similar to that observed at 2 weeks, with increased absolute values in all groups (S2 Fig).

### Histological and histomorphometric findings

Histological analysis demonstrated distinct differences in callus composition among the treatment groups. The PTH monotherapy and PTH → Scl-Ab groups showed a larger cartilage area within the fracture callus, whereas the Scl-Ab monotherapy and Scl-Ab → PTH groups showed more advanced mineralized tissue formation and reduced cartilage content.

**Fig 6:**
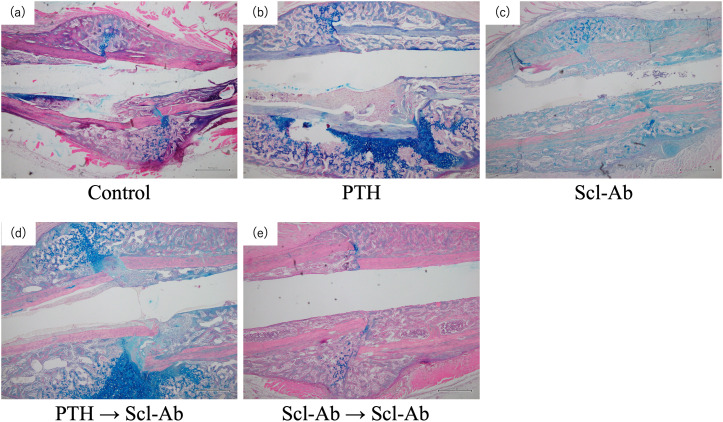
Representative histological sections of fracture callus from each group. Representative histological sections of fracture callus from the Control, PTH, Scl-Ab, PTH → Scl-Ab, and Scl-Ab → PTH groups are shown in panels (a)-(e), respectively. Sections were stained with Alcian blue/hematoxylin and Orange G to visualize cartilage and bone tissue.

**Fig 7:**
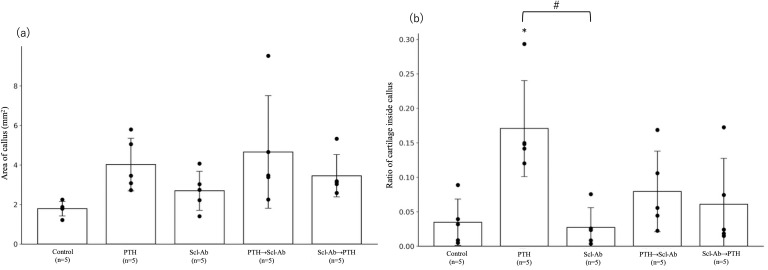
Histomorphometric analysis of fracture callus. (a) Total external callus area. (b) Ratio of cartilaginous tissue within the external callus. * p < 0.05 compared with the Control group; # p < 0.05 between the indicated groups. Scale bars = 500 μm.

Histomorphometric analysis confirmed these differences in callus composition (Figs 6 and 7). These findings suggest that treatment sequence influenced the progression of endochondral ossification during early fracture healing. At the 1-week endpoint, the Control and monotherapy groups showed similar early histological trends (S3 Fig).

## Discussion

The principal finding of this study was that the sequence of anabolic agent administration significantly influenced early fracture healing, even when the same agents, treatment duration, and cumulative exposure were used. Sequential therapy with Scl-Ab followed by PTH resulted in greater mechanical strength and higher callus mineral density than the reverse sequence. These findings indicate that fracture repair is influenced not only by the choice of anabolic agents, but also by the temporal order in which osteogenic signaling pathways are activated during the healing process.

This result was contrary to our initial hypothesis that PTH administration before Scl-Ab would be advantageous, based on the well-established effects of PTH on the proliferation and early differentiation of immature osteoprogenitor cells [16]. Canonical Wnt/β-catenin signaling plays a central role in fracture healing [1,2], and several animal studies have reported enhanced bone repair after Scl-Ab administration [5]. In human clinical trials, however, Scl-Ab has not shown a clear fracture-healing benefit [6,7], whereas PTH analogs have demonstrated more consistent clinical efficacy across several fracture types [11–15]. Although both agents are classified as anabolic therapies, their biological actions differ substantially. PTH stimulates bone turnover through a remodeling-based mechanism and promotes osteoprogenitor proliferation and early differentiation [3,16]. In contrast, Scl-Ab activates Wnt/β-catenin signaling and enhances osteoblastic differentiation and mineralization, with concomitant suppression of bone resorption [3,4].

The findings from the monotherapy groups illustrate these mechanistic differences. PTH treatment significantly increased callus volume, consistent with enhanced cellular proliferation and callus expansion [8,10]. By contrast, Scl-Ab treatment produced higher callus vBMD and superior mechanical strength despite a smaller callus volume, consistent with previous preclinical observations [5,3]. The absence of a significant mechanical advantage in the PTH monotherapy group at postoperative day 14 may reflect the early evaluation time point. At this stage, increased callus volume does not necessarily translate into greater mechanical competence, because callus mineralization and maturation are key determinants of strength. Thus, the present findings are best interpreted as differences in the rate and phase of fracture healing rather than as differences in final healing capacity. It remains possible that PTH monotherapy may provide mechanical advantages at later stages of healing [8,20].

The interpretation of vBMD also differs between non-fractured tibiae and fracture callus. In non-fractured tibiae, trabecular vBMD likely reflects the systemic skeletal response of intact bone to each anabolic regimen. At the fracture site, callus vBMD reflects the mineralization and maturation status of newly formed repair tissue, which is strongly affected by callus composition and the progression of endochondral ossification. In the present study, PTH monotherapy resulted in higher trabecular vBMD than Scl-Ab monotherapy in non-fractured tibiae, whereas Scl-Ab monotherapy resulted in higher callus vBMD than PTH monotherapy at the fracture site. This apparent discrepancy suggests that the systemic anabolic response in intact trabecular bone does not necessarily parallel local callus maturation during fracture repair. The lower callus vBMD observed in the PTH monotherapy and PTH-first sequential therapy groups may be explained, at least in part, by the higher proportion of cartilaginous tissue within the callus, indicating that these groups remained at a relatively earlier stage of endochondral ossification at postoperative day 14.

The superior outcome observed with Scl-Ab-first sequential therapy raises important mechanistic considerations. Previous studies have shown that β-catenin signaling is essential for osteoblastic lineage commitment, whereas insufficient β-catenin activity in immature osteoprogenitor cells favors chondrogenic differentiation [2]. Early activation of Wnt/β-catenin signaling by Scl-Ab may therefore direct progenitor cell fate toward the osteoblastic lineage during a critical early phase of fracture repair, thereby promoting earlier callus mineralization and functional maturation [1,3]. Conversely, when PTH is administered first, enhanced cellular proliferation may occur before sufficient Wnt/β-catenin activation, potentially prolonging the cartilaginous phase of endochondral ossification. This interpretation is consistent with the histological findings showing a higher proportion of cartilaginous tissue in the callus of both the PTH monotherapy and PTH → Scl-Ab groups.

This mechanistic interpretation should be considered hypothesis-generating. The present study did not directly assess Wnt/β-catenin signaling activity, osteogenic gene expression, SOST expression, or lineage-specific markers at the fracture site. The absence of immunohistochemical staining and qPCR analysis is therefore an important limitation. The proposed link between early Scl-Ab administration and enhanced osteoblastic lineage commitment is supported by prior experimental literature [1,2], but it was not directly validated in this study. Other mechanisms, including differences in mineralization kinetics, coupling between callus maturation and mechanical competence, or indirect effects on the fracture microenvironment, may also have contributed to the observed sequence-dependent outcomes.

The discrepancy between favorable preclinical findings and the limited clinical efficacy of Scl-Ab for fracture healing also requires consideration. Several animal studies have demonstrated beneficial effects of Scl-Ab on fracture repair, whereas randomized clinical trials in humans have not shown clear acceleration of fracture healing [6,7]. This difference may be related to species-specific differences in bone remodeling rate, fracture-healing kinetics, and the temporal progression of callus maturation [23]. Clinical trials also rely primarily on radiographic and clinical endpoints, whereas direct biomechanical assessment of the healing fracture is not feasible in humans [24]. The present study highlights the importance of this distinction. PTH monotherapy increased callus volume and may appear favorable on radiographic assessment alone, whereas Scl-Ab monotherapy and Scl-Ab → PTH sequential therapy produced superior mechanical strength. Thus, radiographic callus formation may not fully reflect the mechanical competence of the healing fracture.

Several limitations should be acknowledged. First, fracture healing was evaluated mainly at an early time point. Postoperative day 14 was appropriate for comparing early callus maturation and mechanical competence after completion of the 2-week sequential treatment regimen. The 3-week endpoint showed findings broadly consistent with those at 2 weeks (S1 Fig and S2 Fig). Nevertheless, the present study cannot determine whether the observed sequence-dependent differences persist until final union or influence long-term remodeling. Future studies incorporating earlier and later time points are needed to clarify the temporal progression of healing, final union rates, long-term callus remodeling, and the potential risk of delayed union or non-union after sequential anabolic therapy.

Second, micro-CT analysis of non-fractured tibiae provided supportive evidence of systemic skeletal effects, but systemic bone metabolic and mechanical responses were not comprehensively evaluated. Serum bone turnover markers, dynamic histomorphometry, histological analyses of non-fractured bone such as tartrate-resistant acid phosphatase or osteocalcin staining, and biomechanical testing of intact bone were not performed. Therefore, the non-fractured tibial micro-CT data should be interpreted as structural evidence of systemic skeletal response rather than as a comprehensive assessment of systemic bone metabolism or mechanical competence.

Taken together, the present findings indicate that Scl-Ab–first sequential anabolic therapy promotes earlier callus mineralization and mechanical recovery compared with PTH-first therapy in a murine tibial osteotomy model. These results primarily inform the temporal dynamics of early fracture repair rather than final union or long-term remodeling. From a translational perspective, the temporal orchestration of anabolic signaling may be relevant in clinical scenarios requiring rapid restoration of mechanical stability, such as weight-bearing long-bone fractures or fractures at high risk of delayed union [6,7,11]. While direct extrapolation to humans requires caution, this study highlights a conceptual shift from focusing solely on agent selection toward optimizing the timing and sequence of osteogenic signaling during fracture repair.

## Supporting information

S1 Dataset Raw data used for statistical analyses. (XLSX)

S1 Fig Micro–computed tomography analysis of fracture callus at the 3-week endpoint. (a) Volume of mineralized external callus at the fracture site. (b) Volumetric bone mineral density of the mineralized external callus. * p < 0.05 compared with the Control group; # p < 0.05 between the indicated groups.
(TIFF)

S2 Fig Biomechanical assessment by three-point bending test at 3-week endpoint. (a) Ultimate load to failure. (b) Loading stiffness. * p < 0.05 compared with the Control group; # p < 0.05 between the indicated groups.
(TIFF)

S3 Fig Histological and histomorphometric analysis of fracture callus at 1 week postoperatively. Representative histological sections of fracture callus from the Control, PTH, and Scl-Ab groups at 1 week postoperatively are shown in panels (a)-(c), respectively. Sections were stained with Alcian blue/hematoxylin and Orange G to visualize cartilage and bone tissue. Histomorphometric analysis demonstrated the total external callus area (d) and the ratio of cartilaginous tissue within the external callus (e). Data are presented as mean ± standard deviation with individual data points. *p < 0.05 compared with the Control group; #p < 0.05 between the indicated groups. Scale bars = 500 μm.
(TIFF)
