# Supplementary figures and images for: Sequential administration of sclerostin antibody and parathyroid hormone differentially modulates fracture healing in a murine tibial osteotomy model
Source: PLoS One. 2026 Jul 17;21(7):e0354181. doi: 10.1371/journal.pone.0354181 (PMC13379105; doi:10.1371/journal.pone.0354181)

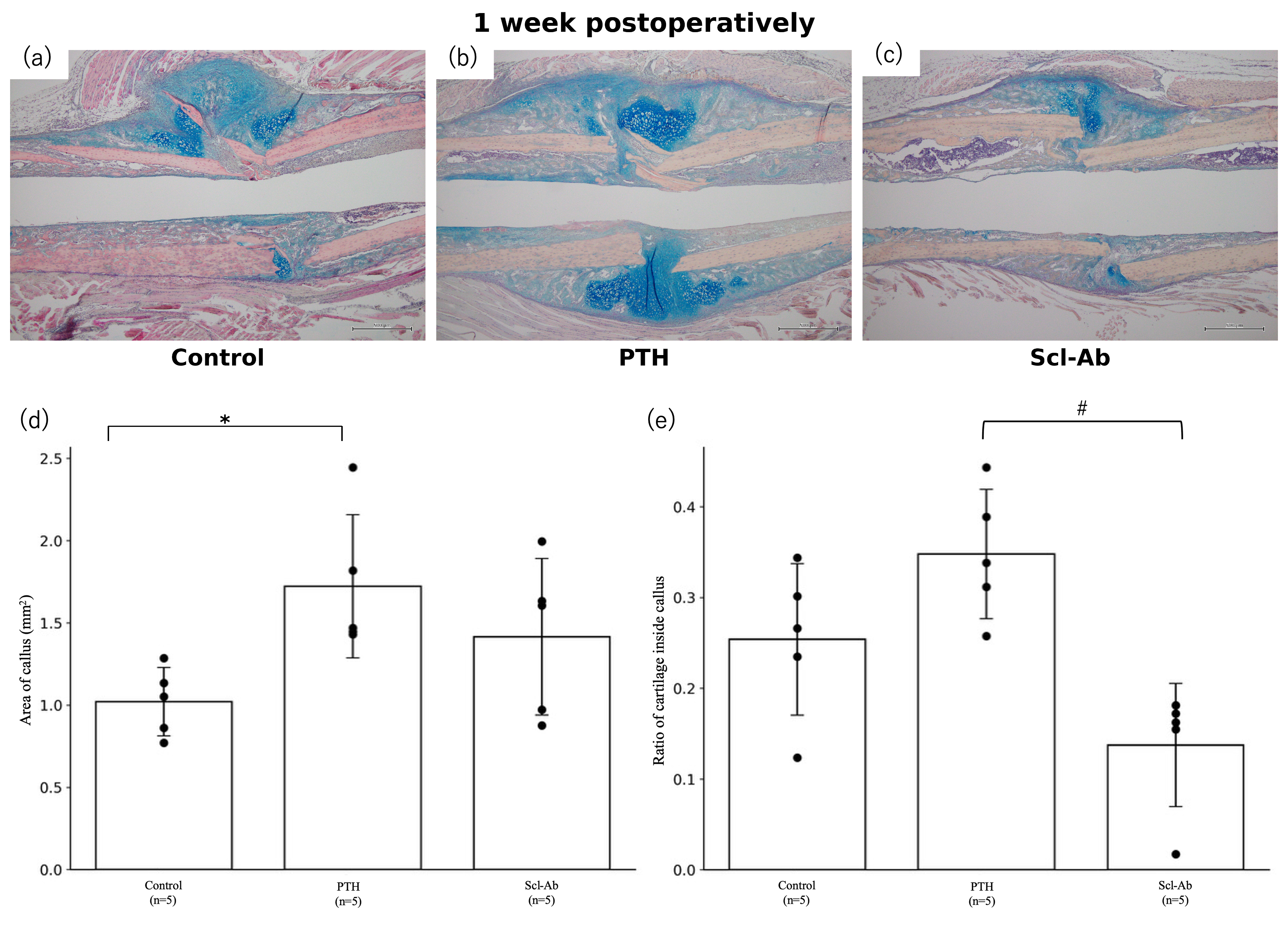

Supplement: S3 Fig — Representative histological sections of fracture callus from the Control, PTH, and Scl-Ab groups at 1 week postoperatively are shown in panels (a)-(c), respectively. Sections were stained with Alcian blue/hematoxylin and Orange G to visualize cartilage and bone tissue. Histomorphometric analysis demonstrated the total external callus area (d) and the ratio of cartilaginous tissue within the external callus (e). Data are presented as mean ± standard deviation with individual data points. *p < 0.05 compared with the Control group; #p < 0.05 between the indicated groups. Scale bars = 500 μm. (TIFF) [file pone.0354181.s004.tiff]
